# The impact of preoperative waiting time in Stage II–III gastric or gastroesophageal junction cancer: A population‐based cohort study

**DOI:** 10.1002/cam4.6320

**Published:** 2023-07-04

**Authors:** Chih‐Chieh Yen, Yi‐Hsin Yang, Hsiu‐Ying Ku, Huang‐Ming Hu, Su‐Shun Lo, Hung‐Chi Chang, Yee Chao, Jen‐Shi Chen, Hsiu‐Po Wang, Tsang‐En Wang, Li‐Yuan Bai, Ming‐Shiang Wu, Chia‐Jui Yen, Li‐Tzong Chen, Yan‐Shen Shan

**Affiliations:** ^1^ Department of Oncology National Cheng Kung University Hospital, College of Medicine, National Cheng Kung University Tainan Taiwan; ^2^ Institute of Clinical Medicine, School of Medicine National Cheng Kung University Tainan Taiwan; ^3^ National Institute of Cancer Research, National Health Research Institutes Tainan Taiwan; ^4^ Department of Healthcare Administration Asia University Taichung Taiwan; ^5^ Department of Internal Medicine Kaohsiung Medical University Hospital, Kaohsiung Medical University Kaohsiung Taiwan; ^6^ Department of Internal Medicine Kaohsiung Municipal Ta‐Tung Hospital Kaohsiung Taiwan; ^7^ Department of Surgery National Yang Ming Chiao Tung University Hospital Yilan Taiwan; ^8^ Department of Surgery Chang‐Hua Christian Hospital Changhua Taiwan; ^9^ Department of Oncology, School of Medicine, Taipei Veterans General Hospital National Yang Ming Chiao Tung University Taipei Taiwan; ^10^ Department of Hematology‐Oncology Linkou Chang Gung Memorial Hospital and Chang Gung University Linkou Taiwan; ^11^ Department of Internal Medicine National Taiwan University College of Medicine and Hospital Taipei Taiwan; ^12^ Department of Internal Medicine Mackay Memorial Hospital Taipei Taiwan; ^13^ Division of Hematology and Oncology, Department of Internal Medicine China Medical University Hospital, and China Medical University Taichung Taiwan; ^14^ Department of Surgery, National Cheng Kung University Hospital College of Medicine, National Cheng Kung University Tainan Taiwan

**Keywords:** gastric cancer, gastroesophageal junction cancer, preoperative waiting time

## Abstract

**Background:**

Gastrectomy remains the curative option in gastric cancer. However, the growing concern that preoperative waiting jeopardizes survival has not been fully addressed. The present population‐based cohort study aimed to clarify the impact of preoperative waiting time (PreWT).

**Methods:**

We included patients with clinical Stage II–III gastric cancer who received curative surgery from 2008 to 2017 of Taiwan Cancer Registry. PreWT was defined as the time from endoscopic diagnosis to surgery. The prognostic impact on overall survival (OS) was evaluated with Cox and restricted cubic spline regressions.

**Results:**

A total of 3059 patients with a median age of 68 years were evaluated. The median PreWT was 16 days (interquartile range, 11–24 days), and patients with a shorter PreWT were younger, had a more advanced disease and received adjuvant therapies. Despite a shorter OS occurring with prolonged PreWT (median OS by PreWT [days]: 7–13, 2.7 years; 14–20, 3.1 years; 21–27, 3.0 years; 28–34, 4.7 years; 35–31, 3.7 years; 42–48, 3.4 years; 49–118, 2.8 years; *p* = 0.029), the differences were not significant after adjustment. The Cox and restricted cubic spline regressions showed that prolonged PreWT was not a significant prognostic factor for OS (*p* = 0.719).

**Conclusions:**

The population‐based study suggests that a PreWT of 49–118 days does not independently correlate with a poor prognosis in Stage II–III gastric cancer. The study provides rationale for a window period for preoperative therapies and patient optimization.

## Abstract 2

The population‐based study suggests that preoperative waiting within an acceptable range dose not impact the survival in Stage II–III gastric or gastroesophageal junction cancer.

## 1 INTRODUCTION

Gastric cancer is one of the most prevalent gastrointestinal malignancies and is the third most common cause of cancer‐related deaths worldwide.^1^ Despite major advances in systemic treatments, surgery remains the only curative option for patients with resectable disease. Multidisciplinary approaches, such as perioperative or neoadjuvant chemotherapies, have been proven to improve both surgical outcomes and long‐term survival in patients with high‐risk or locally advanced disease.^2^, ^3^, ^4^ However, prolonged preoperative delay imposed by these treatments contributes to risk of progression to unresectable disease or ineligibility for surgery owing to treatment‐related toxicities.^5^ In addition, a demand for comprehensive preoperative assessment or intervention has been expanding as the standard of care in recent years.^6^ Such efforts also result in a further delay in curative resection after endoscopic diagnosis. The acceptable interval from endoscopic diagnosis to curative surgery remains largely undetermined and is not routinely described in the international guidelines.

The acceptable preoperative waiting time (PreWT) is an unresolved dilemma because an overshortening of PreWT might be complicated by poor preoperative preparation or conditioning of the patient, while unnecessary lengthening might result in disease progression or psychological stress of the patients.^7^ In the absence of a universal consensus, the impact of PreWT on surgical outcome or prognosis remains to be determined. The United Kingdom Cancer Reform Strategy suggested a standard PreWT of less than 31 days in all cancer patients.^8^ A Korean study by Yun et al. reported that a PreWT of longer than 31 days affected survival in patients with curatively resected gastric cancer in low‐volume hospitals.^9^ However, a study from the Netherlands indicated that a longer PreWT of 35–56 days was not associated with a worse survival.^10^ In addition to the survival impact, PreWT is recognized as a valid indicator for quality, efficiency, and equity of healthcare, which should be carefully accounted for by healthcare administrations and evaluated in nationwide surveillance.^11^

Despite several reports about the survival impact of PreWT in patients with resectable gastric cancer, the clinical significance and acceptable interval of PreWT were inconclusive.^12^, ^13^, ^14^ This could be because many of the results were derived from a single institution and may have had cultural and regional disparities, which might not be applicable elsewhere. A large population‐based study with an adequate follow‐up time is required to address the issue. Furthermore, a detailed elucidation of an exact acceptable value for PreWT may also provide essential information for designing prospective clinical trials and modifying healthcare policies. Therefore, we utilized the population‐based cancer registry to investigate the significance of PreWT for patients with resectable stage II–III gastric or gastroesophageal junction (GEJ) cancer and potentiate future interventional clinical trials.

## 2 MATERIALS AND METHODS

### 2.1 Patients

Patients with resectable gastric or GEJ adenocarcinoma who received surgery between January 2008 and December 2017 in Taiwan were enrolled. Resectable disease is defined as a clinical stage (cStage) IIA–IIIC disease following the American Joint Committee on Cancer staging system version 7.0 at initial diagnosis. All patients had received radical gastrectomy plus ≥D2 lymph node (LN) dissection with intent to cure. Patients who had the following conditions were excluded: (1) a clinical metastatic disease (cStage IV) including peritoneal washing cytology positive for malignant cells at presentation, (2) a recurrent tumor resulting from an antecedent primary disease, (3) an incomplete surgery in which resection margins could not be assessed, (4) Stage I tumors or carcinoma in situ that could potentially be resected by endoscopy, (5) tumors other than adenocarcinoma, such as primary lymphoid or neuroendocrine malignancies, (6) tumors of a primary site other than the stomach or GEJ, (7) receiving preoperative therapies including radiotherapy or chemotherapy, (8) receiving an emergent or overtly delayed surgery with a PreWT <7 or > 118 days, which might contradict the purpose of surgery as optimally curative, and (9) early deaths within 56 days as diagnosis, which excluded the possibility of potential preoperative waiting. Patients who received postoperative adjuvant therapies, such as chemotherapy, radiotherapy, or concurrent chemoradiotherapy (CCRT) were allowed in the study.

### 2.2 Clinical and demographic characteristics

The general practice in Taiwan is to refer patients for a potentially curative treatment after the confirmed diagnosis as soon as possible in any of the National Health Insurance (NHI)‐reimbursed medical institutes. To avoid transferal delays or institutional bias while calculating the PreWT, we only included patients who were diagnosed and received surgery in the same institute. All‐cause mortality is defined as death by any causes. Cancer‐related mortality is defined as death related to the indicated gastric or GEJ cancer. Overall survival (OS) is defined as the time interval between endoscopic diagnosis of the disease to death resulting from either all or cancer‐related causes, which are then discussed separately.

### 2.3 Data source

All information used in the study was retrieved from the Taiwan Cancer Registry (TCR), which is a population‐based cancer registry system established by the Health Promotion Administration, Ministry of Health and Welfare, in Taiwan.^15^, ^16^, ^17^ We have provided introductory and descriptive information on the TCR in Data S1. Briefly, we incorporated the data with the International Classification of Diseases‐O‐3 C160 to C169, encompassing primary gastric or GEJ adenocarcinoma from January 2008 to December 2017.^18^ Clinical and demographic data were retrieved, and we extended the follow‐up period until December 2019 in all included cases. All data had undergone a valid digital de‐identification process and thereby was blinded to all investigators of the study.

### 2.4 Statistical analysis

Clinical and demographic features are presented in descriptive statistics by frequency, percentage, mean and standard deviation, median, and interquartile. We used Student's *t*‐test for comparing continuous variables and chi‐squared test for categorical variables. Survival analysis was estimated by the Kaplan–Meier method and compared by the log–rank test. Univariate and multivariable Cox proportional hazard regression were used to evaluate the prognostic significance of PreWT. We computed the adjusted hazard ratios based on possible confounding factors in the multivariable model, which are described in Table S1.

We also conducted three sensitivity analyses in addition to the primary analysis as follows: (1) inclusion of patients whose PreWT was ≥119 days so that those with extreme PreWT were still included, (2) the prognostic impact of PreWT based on pStage, and (3) evaluation of death events by cancer‐related mortality. The differences of cStage versus pStage in the included patients were also described. In addition to the traditional categorizing of PreWT into weeks, we also incorporated the restricted cubic spline regression approach to confirm the robustness of results and dynamic changes over time under a prespecified reference value.^19^ We presumed the statistically significant level by a two‐tailed α at 0.1. All multiple comparisons were adjusted by Bonferroni correction in the study. We employed SAS® 7.0 (SAS institute Inc.) for data management and computing and GraphPad Prism® 9.1 (GraphPad Software) for graphic production.

## 3 RESULTS

### 3.1 Patients and the distribution of PreWT

**FIGURE 1:**
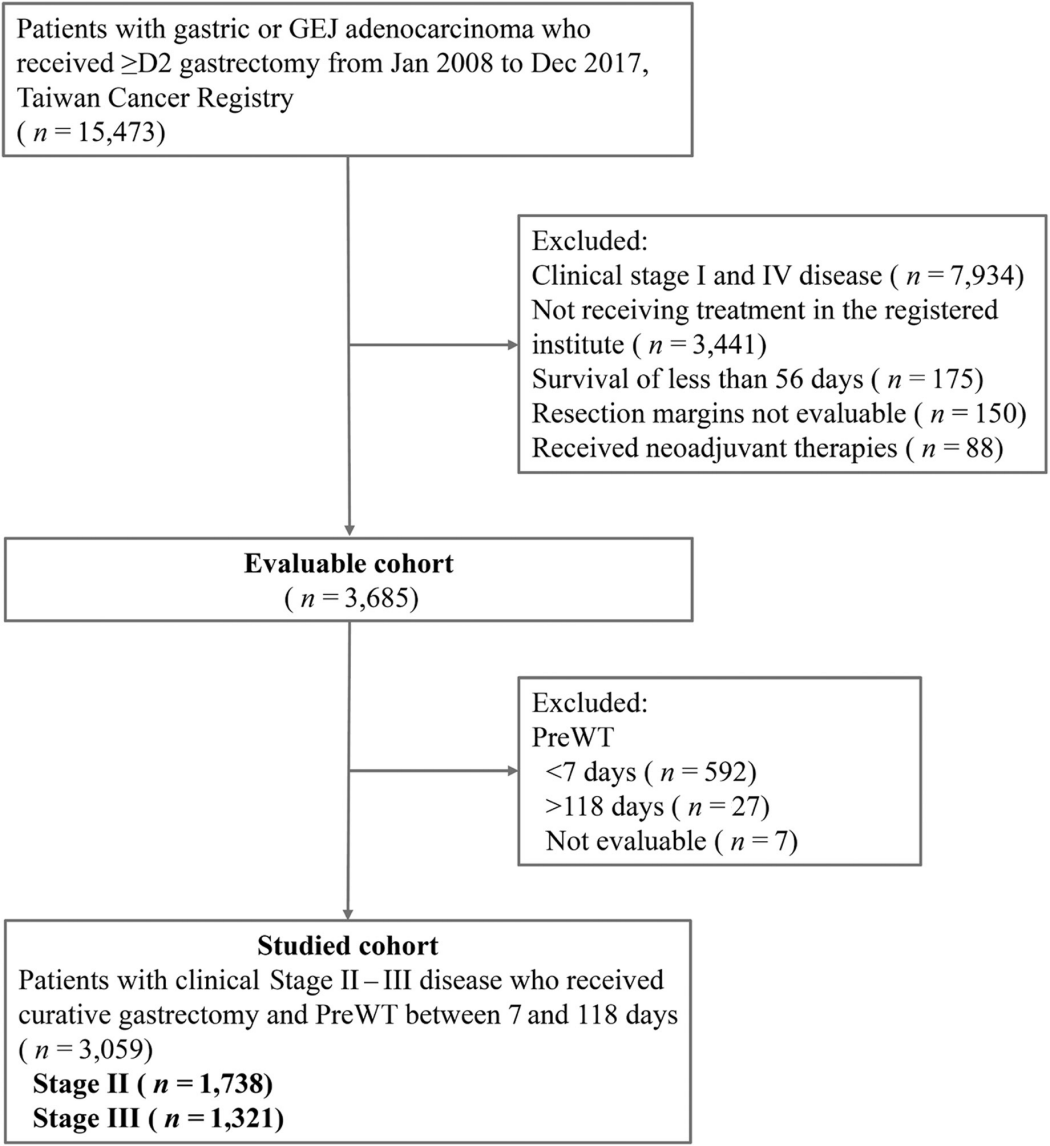
Flow diagram of the study. GEJ, gastroesophageal junction; PreWT, preoperative waiting time.

**FIGURE 2:**
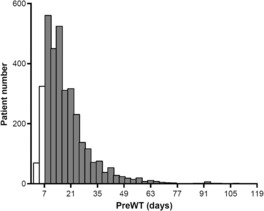
Distribution of the patients. Histogram of the patients by PreWT. The gray bars indicated those with PreWT of 7–118 days and were enrolled in the study. The white bars indicated those with PreWT of <7 days and were not included in the study. PreWT, preoperative waiting time.

A total of 15,473 patients who were diagnosed with gastric or GEJ adenocarcinoma between January 2008 and December 2017 and ≥ D2 gastrectomy were initially screened. From among the evaluable patients (*n* = 3685), we included patients with a PreWT of 7–118 days in the primary analysis (*n* = 3059), out of which 1738 patients had cStage II and 1321 patients had cStage III disease. The flow diagram of the study is provided in Figure 1. In the evaluable cohort, the range of PreWT was 1–314 days. In the studied cohort who had a PreWT between 7 and 118 days, the median time was 16 days with an interquartile range (IQR) of 11–24 days. The histogram of the studied population is shown in Figure 2 and that of those with cStage II or III disease in Figure S1.

### 3.2 Patient characteristics

In the studied cohort, the median age was 68 years (IQR, 58–77 years) with a male predilection (64%) (Table 1). Stage II patients accounted for 57% in the population, and the most common primary tumor sites were the antrum (39%) and body (30%). The surgical results were generally adequate, with 89% of patients having received a dissection of ≥15 LNs and 89% having achieved a margin‐free resection. The majority of the patients had advanced disease (pStage III–IV disease, 64%). Fifty‐two and 11% of the patients had received either adjuvant chemotherapy or radiotherapy, respectively. In general, the characteristics between different PreWT groups were similar and balanced in the studied cohort. However, patients with a shorter PreWT (7–20 days) tended to be younger, had a more advanced disease by pStage, had larger tumor size, had more LNs dissected, and received more adjuvant therapies, as compared to those with a longer PreWT (21–35, 35–48 and 49–118 days). In addition, we observed a discrepancy between cStage versus pStage in the included patients. On clinical to pathological stage progression, 49% of patients with initial cStage II disease had a final pStage of III–IV and 15% with cStage III disease had a pStage of IV. Likewise, patients with a shorter PreWT had a higher risk of stage progression as compared to those with a longer PreWT. The detailed information is provided in Table S2.

**TABLE 1:** Patient characteristics.

	All patients	Patients with a PreWT (days)				
	7–118	7–20	21–34	35–48	49–118	*p*
	(*n* = 3059)	(*n* = 2058)	(*n* = 689)	(*n* = 196)	(*n* = 116)	
Sex, *n* (%)						0.466
Male	1944 (63.6)	1324 (64.3)	430 (62.4)	116 (59.2)	74 (63.8)	
Age						0.002
Median (IQR)	68 (58–77)	68 (58–77)	69 (58–77)	71 (61–80)	71 (61–80)	
Clinical stage, *n* (%)						0.004
II	1738 (56.8)	1128 (54.8)	408 (59.2)	130 (66.3)	72 (62.1)	
III	1321 (43.2)	930 (45.2)	281 (40.8)	66 (33.7)	44 (37.9)	
Pathological stage, *n* (%)						<0.001
I	383 (12.5)	202 (9.8)	111 (16.1)	44 (22.4)	26 (22.4)	
II	728 (23.8)	492 (23.9)	162 (23.5)	47 (24.0)	27 (23.3)	
III	1636 (53.5)	1138 (55.3)	350 (50.8)	95 (48.5)	53 (45.7)	
IV	312 (10.2)	226 (11.0)	66 (9.6)	10 (5.1)	10 (8.6)	
Primary site, *n* (%)						0.272
Cardia	348 (11.4)	225 (10.9)	74 (10.7)	32 (16.3)	17 (14.7)	
Body	917 (30.0)	593 (28.8)	230 (33.4)	56 (28.6)	38 (32.8)	
Antrum	1178 (38.5)	822 (39.9)	249 (36.1)	66 (33.7)	41 (35.3)	
Pylorus	198 (6.5)	132 (6.4)	45 (6.5)	14 (7.1)	7 (6.0)	
GEJ	418 (13.7)	286 (13.9)	91 (13.2)	28 (14.3)	13 (11.2)	
Tumor differentiation, *n* (%)						0.434
Well	969 (31.7)	653 (31.7)	208 (30.2)	71 (36.2)	37 (31.9)	
Poor	2018 (66.0)	1350 (65.6)	470 (68.2)	122 (62.2)	76 (65.5)	
Others^a^	72 (2.4)	55 (2.7)	11 (1.6)	3 (1.5)	3 (2.6)	
Tumor size, *n* (%)						<0.001
<30 mm	721 (23.6)	423 (20.6)	206 (29.9)	60 (30.6)	32 (27.6)	
31–50 mm	936 (30.6)	640 (31.1)	211 (30.6)	56 (28.6)	29 (25.0)	
>50 mm	1335 (43.6)	951 (46.2)	258 (37.4)	74 (37.8)	52 (44.8)	
NM	67 (2.2)	44 (2.1)	14 (2.0)	6 (3.1)	3 (2.6)	
Dissected LNs, *n* (%)						0.008
0–14	325 (10.6)	218 (10.6)	62 (9.0)	24 (12.2)	21 (18.1)	
15–29	1181 (38.6)	800 (38.9)	248 (36.0)	86 (43.9)	47 (40.5)	
≥30	1553 (50.8)	1040 (50.5)	379 (55.0)	86 (43.9)	48 (41.4)	
pT stage, *n* (%)						<0.001
1–2	678 (22.2)	394 (19.1)	186 (27.0)	62 (31.6)	36 (31.0)	
3–4	2381 (77.8)	1664 (80.9)	503 (73.0)	134 (68.4)	80 (69.0)	
pN stage, *n* (%)						0.028
0–1	1150 (49.5)	785 (49.1)	252 (48.6)	72 (54.1)	41 (56.2)	
2–3	1169 (50.3)	812 (50.8)	266 (51.3)	59 (44.4)	32 (43.8)	
NA	5 (0.2)	2 (0.1)	1 (0.2)	2 (1.5)	0 (0.0)	
Received adjuvant chemotherapy, *n* (%)						<0.001
Yes	1602 (52.4)	1118 (54.3)	366 (53.1)	81 41.3)	37 (31.9)	
Received adjuvant radiotherapy, *n* (%)						0.059
Yes	329 (10.8)	243 (11.8)	61 (8.9)	16 (8.2)	9 (7.8)	
Resection margin, *n* (%)						0.218
R0	2706 (88.5)	1816 (88.2)	616 (89.4)	172 (87.8)	102 (87.9)	
R1	323 (10.6)	216 (10.5)	71 (10.3)	24 (12.2)	12 (10.3)	
R2	30 (1.0)	26 (1.3)	2 (0.3)	0 (0)	2 (1.7)	
Treatment site, *n* (%)						<0.001
Tertiary^b^	1718 (56.2)	1086 (52.8)	444 (64.4)	119 (60.7)	69 (59.5)	

Abbreviations: GEJ, gastroesophageal junction; IQR, interquartile range; LN, lymph nodes; NA, not available; NM, not measurable; p, pathological; PreWT, preoperative waiting time.

^a^ Including tumors with undifferentiated and unknown histology classification.

^b^ Refers to the treatment site in a tertiary medical institute.

### 3.3 PreWT and overall survival

**FIGURE 3:**
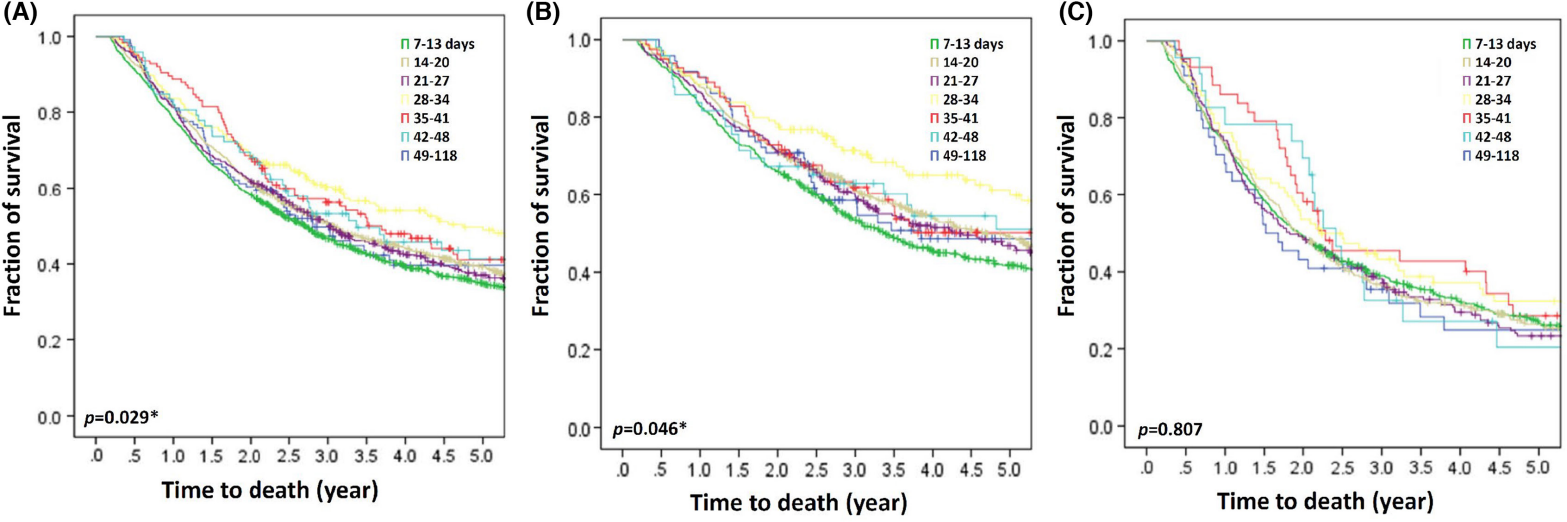
Kaplan–Meier survival analysis. Overall survival results in (A) all, (B) cStage II, and (C) cStage III patients, as stratified by PreWT. Survival differences were compared with log‐rank test. *p < 0.05 as statistically significant.

In the studied cohort, patients with a short PreWT of 7–13 days had shorter OS as compared to those with a PreWT of >14 days (median OS by PreWT [days]: 7–13, 2.7 years; 14–20, 3.1 years; 21–27, 3.0 years; 28–34, 4.7 years; 35–31, 3.7 years; 42–48, 3.4 years; 49–118, 2.8 years; *p* = 0.029) (Figure 3A). Patients with cStage II disease and a PreWT of 7–13 days had a significantly shorter OS as compared to those with a PreWT of >14 days (*p* = 0.046) (Figure 3B). However, patients with cStage III disease and a PreWT of 49–118 days demonstrated a trend of shorter OS, but the differences were not statistically significant (*p* = 0.807) (Figure 3C).

### 3.4 Multivariable Cox and restricted cubic spline regressions

After adjustment for multiple covariates in the Cox regression, PreWT was not statistically significant in patients with cStage II–III disease as an independent prognostic factor. The details of hazard ratios (HRs) and 95% confidence intervals (95% CI) according to different PreWT are shown in Table 2. We observed a reduction in HRs by a PreWT of 35–41 days in all patients (HR = 0.91, 95% CI = 0.66–1.27, *p* = 0.574) and 35–41 days in cStage III patients (HR = 0.80, 95% CI = 0.49–1.30, *p* = 0.368) and an increase by a PreWT of 7–13 days in cStage II patients (HR = 1.37, 95% CI = 0.97–1.94, *p* = 0.075). However, none of these were statistically different as compared to those with a PreWT of 49–118 days.

**TABLE 2:** Hazard ratios for all‐cause mortality.

	All patients (*n* = 3059)						cStage II (*n* = 1738)						cStage III (*n* = 1321)					
PreWT, days	*n*	D	HR^a^	95% CI	*p*	*p* for overall^b^	*n*	D	HR^a^	95% CI	*p*	*p* for overall^b^	*n*	D	HR^a^	95% CI	*p*	*p* for overall^b^
7–13	1200	812	1.21	0.94–1.55	0.132	0.719	641	398	1.37	0.97–1.94	0.075	0.501	559	414	0.91	0.64–1.30	0.613	0.352
14–20	858	557	1.16	0.90–1.49	0.244		487	272	1.25	0.88–1.78	0.213		371	285	0.96	0.67–1.38	0.837	
21–27	476	301	1.26	0.97–1.64	0.082		279	152	1.32	0.91–1.91	0.138		197	149	1.09	0.75–1.60	0.646	
28–34	213	117	0.99	0.73–1.33	0.918		129	58	1.01	0.66–1.53	0.982		84	59	0.89	0.58–1.36	0.580	
35–41	124	73	0.91	0.66–1.27	0.574		81	41	0.99	0.63–1.55	0.948		43	32	0.80	0.49–1.30	0.368	
42–48	72	44	1.07	0.74–1.57	0.713		49	26	1.14	0.69–1.90	0.615		23	18	0.94	0.53–1.67	0.823	
49–118	116	70	1.00				72	36	1.00				44	34	1.00			

Abbreviations: CI, confidence interval; cStage, clinical stage; D, deaths of patients; HR, hazard ratios; n, number of patients; PreWT, preoperative waiting time.

^a^ HRs adjusted for age, sex, treatment site, pathological stage, tumor location, histology grade, size, dissected lymph nodes, resection margin, and receival of adjuvant therapies.

^b^ Overall effect was calculated by multivariable restricted cubic spline regression and compared with Wald test.

**FIGURE 4:**
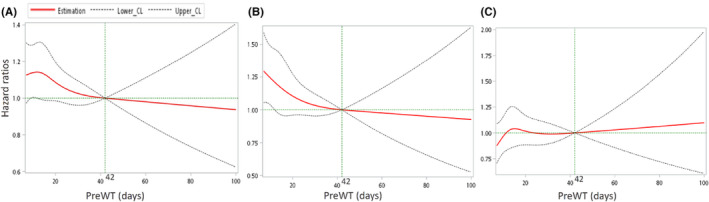
Multivariable restricted cubic spline regression. Multivariable restricted cubic spline plots for HRs of OS in (A) all, (B) stage II, and (C) stage III patients. PreWT of 42 days was demonstrated as the reference value. Red curve showed the estimated HR according to PreWT and dotted lines depicted the 95% of confidence interval within upper and lower levels. All HRs were adjusted for baseline characteristics by multivariable regression and compared with non‐linear Wald test. CL, confidence level; HR, hazard ratio; OS, overall survival; PreWT, preoperative waiting time.

In the restricted cubic spline regression under the reference PreWT of 42 days, we observed a reflection point of HRs at day 14 (HR = 1.14, 95% CI = 0.99–1.30), and the HRs gradually decreased with time in all patients (Figure 4A). In cStage II patients, the HRs also decreased with time when the PreWT was prolonged (Figure 4B). To the contrary, the HRs were approximately constant before day 49 and gradually increased with time in cStage III patients (Figure 4C). However, after adjusting for multiple covariates, PreWT was not a statistically significant prognostic factor with time in patients with cStage II–III disease (*p* = 0.719 in all, *p* = 0.501 in stage II and *p* = 0.352 in stage III patients by the Wald test).

### 3.5 Prognostic factors in the studied cohort

In the studied cohort, univariate Cox regression revealed that receiving adjuvant chemotherapy, disease stage, number of dissected LNs, margin‐free resection, patient age, tumor size, tumor differentiation, treatment site, and primary tumor site were significant prognostic factors for OS in all‐cause mortality. In the multivariable analysis, receiving adjuvant chemotherapy, disease stage, number of dissected LNs, margin‐free resection, patient aged ≥55 years, tumor size, tumor differentiation, treatment site, and sex were statistically significant prognostic factors for OS in all‐cause mortality. Conversely, PreWT was not a statistically significant prognostic factor for OS. The details of HRs, 95% CI, and significant levels are shown in Table 3.

**TABLE 3:** Prognostic factors for all‐cause mortality using Cox proportional regression.

		Univariate regression			Multivariable regression		
	*n*	HR	95% CI	*p*	HR	95% CI	*p*
Adjuvant chemotherapy							
No	1457	1.00					
Yes	1602	0.83	0.76–0.91	<0.001	0.60	0.54–0.68	<0.001
pStage							
I	383	1.00					
II	728	1.87	1.51–2.31	<0.001	2.18	1.75–2.71	<0.001
III	1636	4.39	3.62–5.32	<0.001	5.26	4.24–6.53	<0.001
IV	312	8.52	6.85–10.60	<0.001	6.58	5.21–8.32	<0.001
cStage							
II	1738	1.00					
III	1321	0.76	0.73–0.80	<0.001	0.82	0.75–0.91	<0.001
Dissected LNs							
0–14	325	1.00					
15–29	1181	0.86	0.75–1.00	0.048	0.75	0.65–0.87	<0.001
≥ 30	1553	0.78	0.67–0.90	<0.001	0.62	0.53–0.72	<0.001
Resection margin							
R0	2706	1.00					
R1	323	2.47	2.17–2.80	<0.001	1.78	1.56–2.03	<0.001
R2	30	2.61	1.78–3.81	<0.001	1.47	1.00–2.16	0.053
Age, years							
< 55	511	1.00					
55–74	1517	1.12	0.98–1.28	0.090	1.23	1.08–1.41	0.002
≥ 75	1031	1.82	1.59–2.08	<0.001	1.86	1.61–2.15	<0.001
Tumor size, mm							
< 30	721	1.00					
31–50	936	1.74	1.53–1.99	<0.001	1.18	1.03–1.36	0.021
> 50	1335	2.24	1.97–2.53	<0.001	1.24	1.08–1.42	0.002
NM	67	2.89	2.17–3.85	<0.001	1.68	1.25–2.27	0.001
Tumor differentiation							
Well	969	1.00	1.13–1.38	<0.001	1.15	1.04–1.28	0.006
Poor	2018	1.25					
Others	72	1.44	1.09–1.90	0.010	1.20	0.91–1.60	0.203
Treatment site							
Tertiary	1718	1.00					
Non‐tertiary	1341	1.17	1.07–1.28	0.001	1.15	1.05–1.26	0.003
Sex							
Male	1944	1.00					
Female	1115	0.91	0.83–1.00	0.057	0.91	0.83–1.00	0.048
Adjuvant radiotherapy							
No	2730	1.00					
Yes	329	1.11	0.97–1.27	0.137	1.10	0.94–1.27	0.237
Primary site							
Cardia	348	1.00					
Body	917	0.74	0.63–0.56	<0.001	1.05	0.90–1.22	0.563
Antrum	1178	0.84	0.72–0.97	0.016	1.09	0.94–1.26	0.267
Pylorus	198	0.91	0.73–1.12	0.346	1.12	0.91–1.38	0.295
GEJ	418	1.15	0.97–1.36	0.102	1.21	1.02–1.44	0.030
PreWT, days							
49–118	116	1.00					
7–20	2058	1.07	0.84–1.36	0.605	1.05	0.82–1.34	0.693
21–34	689	0.93	0.73–1.20	0.596	1.09	0.85–1.41	0.491
35–48	196	0.90	0.67–1.20	0.463	0.93	0.69–1.25	0.637

Abbreviations: 95% CI; 95% confidence interval; GEJ, gastroesophageal junction; HR, hazard ratio; LN, lymph nodes; NM, not measurable; PreWT, preoperative waiting time; pStage, pathological stage.

### 3.6 Sensitivity analyses

We performed three sensitivity analyses in the study. First, we included patients with a PreWT ≥119 days (PreWT of 7 to unlimited days) and conducted a multivariable restricted cubic spline regression. The results corresponded to that of the primary analysis, indicating that PreWT was not a statistically significant prognostic factor with time in patients with cStage II–III gastric or GEJ cancer (*p* = 0.707 by Wald test) (Figure S2). Second, we incorporated cancer‐related instead of all‐cause mortality as the defining events for OS and conducted the multivariable Cox regression (Table S3). The results were like those of the primary analysis. None of these were statistically different as compared to those with a PreWT of 49–118 days after adjusting with the covariates. Third, we assessed patients with pStage II–III disease rather than cStage for analysis. It showed similar results that PreWT of 49–118 days was not an independent prognostic factor, as defined either by initial cStage or final pStage (Table S4).

## 4 DISCUSSION

To the best of our knowledge, the present study is one of the largest population‐based cohort studies incorporating real‐world evidence on the prognostic impact of PreWT in patients with resectable gastric or GEJ cancer. The study indicated that a PreWT of 49–118 days did not independently correlate with a poor prognosis as compared to a PreWT less than 49 days.

Our results agreed with those of previous studies. Furukawa et al. reported that a PreWT up to 90 days did not affect the survival in patients with stage II–III gastric cancer.^13^ Han et al. compared the influence of PreWT in patients with resectable gastric or lung cancer and concluded that the former was unaffected by PreWT in terms of mortality.^20^ Brenkman et al.'s Netherlands‐based study, which included the largest Western patient population in the literature, reported that a PreWT of more than 56 days was not associated with an adverse survival outcome as compared to a PreWT less than 35 days.^10^ A similar finding was also reported by Kumazu et al., demonstrating that patients had a comparable OS even when PreWT exceeding 63 days.^14^ However, we still observed a trend of shorter OS in patients with a prolonged PreWT, despite the difference not being statistically significant after adjusting for other prognostic factors. The present study confirmed that a prolonged PreWT within an acceptable range was not critical and decisive in terms of prognostic impact in patients with resectable gastric cancer. Nevertheless, our results did not imply that curative surgery could be unconditionally delayed. Weighing the disease risk and therapeutic benefit is still an essential issue when preoperative delay is inevitable in real‐world clinical practice.

In light of the success of neoadjuvant or perioperative therapies, the frontline treatment paradigm in gastric cancer has changed in the recent decade.^21^, ^22^, ^23^ In prospective phase III trials, a therapeutic interval of at least 56–63 days was required prior to surgery.^2^, ^3^, ^4^, ^24^, ^25^ Our results revealed that an interval of 49–118 days might be a feasible window period for a comprehensive preoperative assessment and allow time to optimize the condition of the patients without concerns that waiting time might jeopardize survival outcome. In addition, we observed a dynamic change in serial HRs when the PreWT was prolonged by 7 days. The risk of mortality was higher in patients with a PreWT of 7–27 days (adjusted HR = 1.26, 95% CI 0.97–1.64, *p* = 0.082) and was the least for patients with a PreWT of 35–41 days (adjusted HR = 0.91, 95% CI 0.66–1.27, *p* = 0.574) as compared to those with a longer PreWT, despite the OS differences not reaching statistical significance. The risk of mortality was also higher in patients with a PreWT less than 30–35 days in the Dutch and Japanese study, consistent with our results.^10^, ^13^ Indeed, an over‐shortening of PreWT might be subjected to result in inadequate preoperative preparation, while a redundant waiting might expose patients to risk of disease progression. Our results echoed a potential optimal preoperative waiting period for clinicians in real‐world practices.

The median PreWT was 16 days from endoscopic diagnosis to curative surgery in the current study, which was relatively shorter than that in previous studies reporting a median time ranging from 31 to 45 days.^9^, ^10^, ^13^, ^14^ The possible explanation was the high accessibility to surgery, reliable efficiency, and abundance of medical facilities in Taiwan.^26^, ^27^, ^28^ The UK healthcare administration introduced a waiting time goal of less than 31 days as a quality indicator for excellent clinical service.^8^, ^29^ Similar appeals were also noted by European, Canadian, and US medical societies for various cancer surgeries.^30^, ^31^, ^32^ Bilimoria et al. reported that 39% of patients with gastric cancer received primary treatment within 30 days from diagnosis, with a median time of 19 days, when the patient was diagnosed and treated at the same hospital in the United States.^33^ Another alternative reason was that we excluded patients who were not diagnosed and operated in the same institute to avoid referral time bias; consequently, iatrogenic referral delays were not accounted in the study. In addition to disease concerns, a prolonged waiting time has been proposed to impose a tremendous psychological burden on patients. Song et al. revealed that anxiety while waiting caused complex mental health consequences.^34^ Although delays in non‐emergent cancer surgeries did not impact survival outcomes, they may decrease patient satisfaction and quality of life.^7^, ^35^, ^36^

We observed differences in patient characteristic based on PreWT. Those with a shorter PreWT represented the patient population that was younger, had a more advanced disease, received a deeper level of LN dissection, and was eligible for adjuvant therapies. To the contrary, elderly unfit patients who had a less advanced disease and demanded more preoperative conditioning delayed the surgery and thus contributed to a longer PreWT. Such findings were compatible with those of other retrospective studies.^9^, ^10^, ^12^, ^14^ Furthermore, patients with a shorter PreWT had a higher risk of underestimated disease burden, which was observed as the inconsistency between initial clinical and final pathological staging. The “stage progression” phenomenon highlighted that those with inadequate preoperative assessment had an underestimated disease extent.

The merits of the present study are relatively adequate case numbers and statistical power in a population‐based design with long follow‐up time. To date, it is the second largest population‐based study following the Korean report by Yun et al.^9^ In addition, we used a valid and high‐quality cancer registry system to derive analyses beyond a single institute. One limitation was that we excluded those with an extreme PreWT to prevent the influences of outliers. However, we still attempted to conduct sensitivity analyses to confirm the robustness of interpreting results and detailed adjustments on multiple confounding factors for prognostic impacts. Both initial clinical and final pathological staging of the disease were accounted for, and the results reflected direct clinical scenarios encountered by practicing clinicians.

## 5 CONCLUSION

The present population‐based cohort study suggests that a PreWT of 49–118 days does not independently correlate with a poor prognosis as compared to a PreWT less than 49 days in patients with stage II–III gastric or GEJ cancer eligible for curative surgery. The results confirm that a waiting time within an acceptable range is not decisive on prognosis. The study provides a rationale for defining a window period for preoperative neoadjuvant therapies.

## AUTHOR CONTRIBUTIONS

**Chi‐Chieh Yen:** Conceptualization (equal); writing – original draft (equal). **Yi‐Hsin Yang:** Conceptualization (equal); formal analysis (equal); writing – original draft (equal). **Hsiu‐Ying Ku:** Data curation (equal); formal analysis (equal). **Huang‐Ming Hu:** Data curation (equal); formal analysis (equal). **Su‐Shun Lo:** Conceptualization (equal); investigation (equal). **Hung‐Chi Chang:** Conceptualization (equal); investigation (equal). **Yee Chao:** Conceptualization (equal); investigation (equal). **Jen‐Shi Chen:** Conceptualization (equal); investigation (equal). **Hsiu‐Po Wnag:** Conceptualization (equal); investigation (equal). **Tsang‐En Wang:** Conceptualization (equal); investigation (equal). **Li‐Yuan Bai:** Conceptualization (equal); investigation (equal). **Ming‐Shiang Wu:** Conceptualization (equal); investigation (equal). **Chia‐Jui Yen:** Conceptualization (equal); investigation (equal). **Li‐Tzong Chen:** Conceptualization (equal); resources (equal). **Yan‐Shen Shan:** Conceptualization (equal); investigation (equal); resources (equal); writing – review and editing (equal).

## FUNDING INFORMATION

This work was funded by the Health Promotion Administration, Ministry of Health and Welfare, Grant no. A1081116. Funded by Tobacco Health and Welfare Taxation. The content of this research may not represent the opinion of the Health Promotion Administration, Ministry of Health and Welfare.

## CONFLICT OF INTEREST STATEMENT

The authors have no conflict of interest to declare.

## ETHICS STATEMENT

This is a retrospective observational population‐based study including patients with standard medical care. All analyzed information underwent a valid de‐identification process in TCR and were blinded to all investigators. Therefore, informed consent was waived. The study is approved by the Institutional Review Board (IRB) at National Cheng Kung University Hospital, Tainan, Taiwan with registry serial number A‐EX‐110‐027 in accordance with the Declaration of Helsinki and its amendments.

## Supporting information


**Data S1.** Supporting Information

## Data Availability

The data that support the findings of this study are available from TCR. However, restrictions apply to their availability upon formal application to the access of electronic recorded data. Researchers are required to delineate reasonable request and be commissioned by the administrative agency of Taiwan to access TCR under specific permissions. Taiwan Cancer Registry (TCR): P.O. Box 84‐310, Taipei, Taiwan (ROC). +886‐2‐23512024. http://tcr.cph.ntu.edu.tw
