# Supplementary material for: The impact of preoperative waiting time in Stage II–III gastric or gastroesophageal junction cancer: A population‐based cohort study
Source: Cancer Med. 2023 Jul 4;12(16):16906–17. doi: 10.1002/cam4.6320 (PMC10501262; doi:10.1002/cam4.6320)
Supplement: Supplementary file 1 — Data S1. Supporting Information [file CAM4-12-16906-s001.pdf]

Supplementary Materials

The Impact of Preoperative Waiting Time in Stage II to III Gastric or Gastroesophageal Junction Cancer: A Population-based Cohort Study

Corresponding author: Yan-Shen Shan, MD. PhD. ysshan@mail.ncku.edu.tw

Data ···· 2

Table 1···· 4

Table 2···· 5

Table 3·· 7

Table 4·· 8

Figure 1···· 9

Figure 2···· 10

Supplementary Data: Taiwan Cancer Registry

Brief introduction

Taiwan Cancer Registry (TCR) is a nationwide population-based cancer registry system established since 1979 in Taiwan. The system records clinical, therapeutic and demographic information on patients with newly diagnosed malignancies in cancer-treating hospitals in Taiwan. All registered medical institutes have affiliated collaborations with National Health Insurance (NHI), which is the only public-based and generalized single-payer medical welfare system in Taiwan. TCR is recognized as one of the high-quality cancer registries in the world and has been harnessed for generating healthcare policies and academic researches [1].

Contents of recording

After several revisions, the database currently includes patient demographics, cancer diagnosis, staging, treatment, recurrence/relapse events, cancer-specific factors/risks, laboratory measurements, survival and events of specific study interests. The detailed and complete version, or the "Long Form", contains 115 items for recording, while the simplified "Short Form" has 45 items, respectively.

Registry party

According to the regulations of NHI-Taiwan and Ministry of Health and Welfare, all medical institutes which contain more than 50 hospital beds and provide cancer-treating services are required to record cancer-related information on TCR, either by paper-based or online documentation, on a seasonal basis. Nearly ninety percent of the incident cancer cases receive diagnostic and therapeutic care in the medical institutes which are registered with TCR in 2017. In addition, annual reports on data validation, quality and completeness are mandatory on both the registry party and database competent organizer upon independent third-party auditing. The Health and Welfare Data Science Center links TCR with other healthcare or demographic theme databases to enhance the spectrum of utility on policy-making and high-quality academic research in Taiwan.

Data validation and quality

TCR incorporates valid quality assessment according to the international guidelines for population-based healthcare registry. The following quality indicators have been implemented on TCR: the completeness on registry data (completeness, %), which delineates the effort to eliminate missing values; percentage of morphologically verified cases (MV, %), representing the precise cancer diagnosis generated from

microscopic identifications; mortality verse incidence ratio (M/I, %), indicating the relationship of incident and mortal cases per year; percentage of death certificate only cases (DCO, %), the number of cases with information limited to death events only. TCR reported the completeness of 98.3%, MV of 93.3%, M/I of 43.3% and DCO of 0.8% in 2017, which is compatible to the international standards, respectively [2].

Timeliness

The timeliness refers to the delay between diagnosis to when the case is being reported. TCR reports the timeliness of 14 months in 2017, which is comparable to the international population-based registry systems.

Considerations on individual identification

Owing to ethical considerations, all information recorded to TCR must receive a valid digital de-identification process. Each patient is therefore given a unique identity code limited of use within the registry system. To prevent potential violations on patient privacy, TCR is blinded to database organizer or investigators of whatever study interests.

Other information

http://tcr.cph.ntu.edu.tw/main.php?Page=N1

TSCR 台灣癌症登記學會
TAIWAN SOCIETY OF CANCER REGISTRY

1. Chiang C-J, Wang Y-W, Lee W-C. Taiwan's Nationwide Cancer Registry System of 40 years: Past, present, and future. Journal of the Formosan Medical Association. 2019 02/01;118.
2. Chiang CJ, You SL, Chen CJ, et al. Quality assessment and improvement of nationwide cancer registration system in Taiwan: a review. Japanese journal of clinical oncology. 2015 Mar;45(3):291-6.

Supplementary Table 1: Baseline characteristics included in the multivariable Cox regression

Variable	Description	Category
Adjuvant therapies	Receival of adjuvant radiotherapy or chemotherapy postoperatively	Adjuvant radiotherapy, chemotherapy or none
Age	Age at diagnosis	<55, 55-74 or ≥75 years of age
Dissected LNs	Number of dissected LNs	<14, 15-29 or ≥30 LNs
Histology grade	Histology differentiation	Well, poor and others
Pathological stage	Pathological tumor (T) and LN stage (N)	T1, T2, T3 or T4; N0, N1, N2 or N3
R0 resection	Margin-free (R0), microscopically (R1) or macroscopically residual resection (R2)	R0, R1 or R2
Sex	Patient sex	Male or female
Treatment site	Scale of the treating medical institute[a]	Tertiary or non- tertiary institute
Tumor location	Primary tumor location at stomach or GEJ	Cardia, antrum, pylorus or GEJ
Tumor size	Maximal diameter of the tumor	<30, 31-50, >50 mm or not measurable

a. The treatment site is graded as follows: primary local/community-based hospitals or clinics, secondary general teaching hospitals, and tertiary medical centers.

LN, lymph node; GEJ, gastroesophageal junction.

Supplementary Table 2: Clinical versus pathological staging and the PreWT in the included patients

	**All patients**		**Patients with a PreWT (days)**								***p***	**PreWT, median (IQR), days**	
	**7-118**		**7-20**		**21-34**		**35-48**		**49-118**				
	**(*n*=3059)**		**(*n*=2058)**		**(*n*=689)**		**(*n*=196)**		**(*n*=116)**				
**Clinical stage, *n* (%)**											0.004		
**II**	**1,738**	**(56.8)**	**1,128**	**(54.8)**	**408**	**(59.2)**	**130**	**(66.3)**	**72**	**(62.1)**			
Pathological stage													
I	332	(19.1)	170	(15.1)	99	(24.3)	40	(30.8)	23	(31.9)	<0.001	20	(14-30)
II	561	(32.3)	376	(33.3)	128	(31.4)	34	(26.2)	23	(31.9)	0.398	16	(11-23)
III	730	(42.0)	499	(44.2)	159	(39.0)	51	(39.2)	21	(29.2)	0.028	15	(11-22)
IV	115	(6.6)	83	(7.4)	22	(5.4)	5	(3.8)	5	(6.9)	0.305	14	(10-21)
Stage progression (+)[a]	845	(48.6)	582	(51.6)	181	(44.4)	56	(43.1)	26	(36.1)	0.004		
**III**	**1,321**	**(43.2)**	**930**	**(45.2)**	**681**	**(40.8)**	**66**	**(33.7)**	**44**	**(37.9)**			
Pathological stage													
I	51	(3.9)	32	(3.4)	12	(4.3)	4	(6.1)	3	(6.8)	0.035	17	(10-25)
II	167	(12.6)	116	(12.5)	34	(12.1)	13	(19.7)	4	(9.1)	<0.001	14	(11-24)
III	906	(68.6)	639	(68.7)	191	(68.0)	44	(66.7)	32	(72.7)	<0.001	14	(11-22)
IV	197	(14.9)	143	(15.4)	44	(15.7)	5	(7.6)	5	(11.4)	<0.001	15	(10-21)
Stage progression (+)[b]	197	(14.9)	143	(15.4)	44	(15.7)	5	(7.6)	5	(11.4)	<0.001		

a. defined as patients with initial cStage II disease who had a final pStage III or IV disease.

b. defined as patients with initial cStage III disease who had a final pStage IV disease.

Supplementary Table 3: Hazard ratios for cancer-specific mortality

	All patients						cStage II						cStage III					
PreWT, days	N	D	HR[a]	95% CI	*p*	*p* for overall[b]	N	D	HR[a]	95% CI	*p*	*p* for overall[b]	N	D	HR[a]	95% CI	*p*	*p* for overall[b]
7-13	1200	623	1.04	0.78-1.39	0.77	0.587	641	295	1.17	0.76-1.78	0.48	0.614	559	328	0.88	0.59-1.31	0.53	0.281
14-20	858	415	1.03	0.77-1.38	0.83		487	187	1.06	0.69-1.63	0.79		371	228	0.96	0.64-1.43	0.83	
21-27	476	216	1.07	0.79-1.45	0.68		279	107	1.18	0.75-1.85	0.47		197	109	0.93	0.60-1.42	0.72	
28-34	213	84	0.97	0.68-1.37	0.85		129	38	0.91	0.54-1.52	0.71		84	46	0.96	0.60-1.56	0.88	
35-41	124	47	0.73	0.49-1.08	0.12		81	26	0.79	0.45-1.39	0.41		43	21	0.61	0.34-1.09	0.09	
42-48	72	25	0.78	0.49-1.27	0.32		49	14	0.71	0.36-1.37	0.30		23	11	0.87	0.43-1.77	0.70	
49-118	116	51	1				72	24	1				44	27	1			

a. HRs adjusted for age, sex, treatment site, pathological stage, tumor location, histology grade, size, dissected lymph nodes, resection margin and receival of adjuvant therapies.

b. Overall effect was calculated by multivariable restricted cubic spline regression and compared with Wald test.

PreWT, preoperative waiting time; n, number of patients; D, deaths of patients; HR, hazard ratios, CI, confidence interval.

Supplementary Table 4: Hazard ratios by pathological staging

	All patients						pStage II						pStage III					
PreWT, days	N	D	HR[a]	95% CI	*p*	*p* for overall[b]	N	D	HR[a]	95% CI	*p*	*p* for overall[b]	N	D	HR[a]	95% CI	*p*	*p* for overall[b]
7-13	1,328	900	0.92	0.75-1.14	0.447	0.646	470	252	0.82	0.57-1.18	0.292	0.608	858	648	0.92	0.71-1.19	0.527	0.829
14-20	954	606	0.89	0.72-1.10	0.282		368	171	0.73	0.50-1.06	0.096		586	435	0.92	0.70-1.19	0.514	
21-27	531	330	0.92	0.74-1.16	0.486		198	86	0.72	0.48-1.08	0.110		333	244	0.96	0.73-1.27	0.795	
28-34	254	139	0.86	0.66-1.11	0.247		119	46	0.72	0.46-1.13	0.151		135	93	0.88	0.64-1.22	0.454	
35-41	139	95	0.78	0.59-1.04	0.089		54	26	0.77	0.46-1.29	0.325		85	69	0.77	0.54-1.08	0.128	
42-48	83	55	0.93	0.67-1.29	0.650		32	17	0.91	0.51-1.64	0.755		51	38	0.85	0.57-1.27	0.425	
49-118	136	99	1.00				59	35	1.00				77	64	1.00			

a. HRs adjusted for age, sex, treatment site, pathological stage, tumor location, histology grade, size, dissected lymph nodes, resection margin and receival of adjuvant therapies.

b. Overall effect was calculated by multivariable restricted cubic spline regression and compared with Wald test.

PreWT, preoperative waiting time; n, number of patients; D, deaths of patients; HR, hazard ratios, CI, confidence interval.

Supplementary Figure 1: distribution of the patients by stage

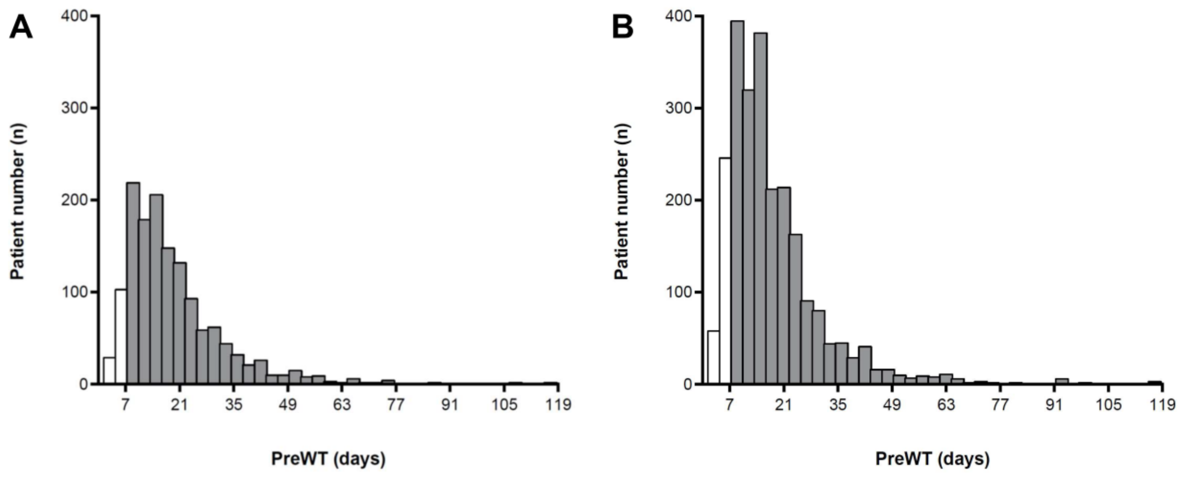

Histogram by PreWT in (A) stage II and (B) stage III patients. The gray bars indicated those with a PreWT between 7 to 118 days and were enrolled in the study. The white bars indicated those with PreWT of <7 days and were not included in the study.

PreWT, preoperative waiting time.

Supplementary Figure 2: multivariable restricted cubic spline regression including patients with a PreWT ≥119 days

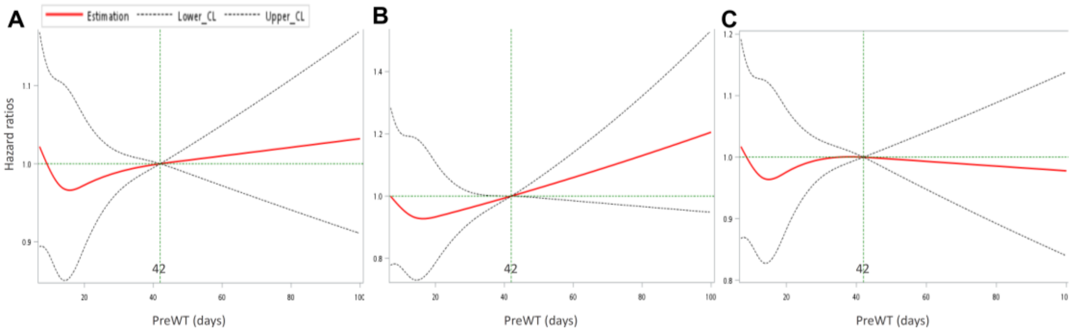

Multivariable restricted cubic spline plots for HRs of OS in (A) all, (B) stage II and (C) stage III patients. PreWT of 42 days was demonstrated as the reference value. Red curve showed the estimated HR according to PreWT and dotted lines depicted the 95% of confidence interval within upper and lower level. All HRs were adjusted for baseline characteristics by multivariable regression and compared with non-linear Wald test.

HR, hazard ratio; OS, overall survival; PreWT, preoperative waiting time; CL, confidence level.
